# Low-Dosage Inhibition of Dll4 Signaling Promotes Wound Healing by Inducing Functional Neo-Angiogenesis

**DOI:** 10.1371/journal.pone.0029863

**Published:** 2012-01-18

**Authors:** Alexandre Trindade, Dusan Djokovic, Joana Gigante, Marina Badenes, Ana-Rita Pedrosa, Ana-Carina Fernandes, Luís Lopes-da-Costa, Valery Krasnoperov, Ren Liu, Parkash S. Gill, António Duarte

**Affiliations:** 1 Centro Interdisciplinar de Investigação em Sanidade Animal (CIISA), Lisbon Technical University, Lisbon, Portugal; 2 Instituto Gulbenkian de Ciência, Oeiras, Portugal; 3 Vasgene Therapeutics, Los Angeles, California, United States of America; 4 Department of Pathology, University of Southern California, Los Angeles, California, United States of America; 5 Department of Medicine, University of Southern California, Los Angeles, California, United States of America; Feinberg Cardiovascular Research Institute, Northwestern University, United States of America

## Abstract

Recent findings regarding Dll4 function in physiological and pathological conditions indicate that this Notch ligand may constitute an important therapeutic target. Dll4 appears to be a major anti-angiogenic agent, occupying a central role in various angiogenic pathways. The first trials of anti-Dll4 therapy in mice demonstrated a paradoxical effect, as it reduced tumor perfusion and growth despite leading to an increase in vascular density. This is seen as the result of insufficient maturation of the newly formed vasculature causing a circulatory defect and increased tumor hypoxia. As Dll4 function is known to be closely dependent on expression levels, we envisioned that the therapeutic anti-Dll4 dosage could be modulated to result in the increase of adequately functional blood vessels. This would be useful in conditions where vascular function is a limiting factor for recovery, like wound healing and tissue hypoxia, especially in diabetic patients. Our experimental results in mice confirmed this possibility, revealing that low dosage inhibition of Dll4/Notch signaling causes improved vascular function and accelerated wound healing.

## Introduction

Wound healing is a physiological process required for maintenance of an intact skin barrier. Angiogenesis, the growth of new blood vessels, is an important natural process required for healing wounds and for restoring blood flow to tissues after injury or insult [1]. Following a traumatic injury, angiogenesis is initiated by multiple molecular signals, including hemostatic factors, inflammation, cytokine growth factors, and cell-matrix interactions, and is mediated throughout the entire wound-healing process [2], [3]. New blood vessels grow via a cascade of biological events to form granulation tissue in the wound bed [4]. This process is sustained until the terminal stages of healing, when angiogenesis is slowed by reduced levels of growth factors, resolution of inflammation, stabilized tissue matrix, and endogenous inhibitors of angiogenesis [2], [3]. Defects in the angiogenesis pathway impair granulation and delay healing [4].

Because angiogenesis is required for wound healing, its induction is beneficial in many clinical situations to achieve wound closure, enhance tissue vascularization, improve local circulation, and promote healing and regeneration. This capability is controlled by pro- and anti-angiogenic factors present throughout the body. A precise physiological balance exists between pro-angiogenic and endogenous anti-angiogenic inhibitors, such that vascular growth is normally suppressed. Immediately following injury, however, pro-angiogenic factors are released into the wound bed, and a shift occurs in the balance of regulators favoring vascular growth [2], [3], [5], [6], [7].

There are already a few pro-angiogenic therapies available for use in the treatment of wounds. The first drug of this kind was the recombinant human platelet-derived growth factor-BB (rhPDGF-BB), which produces good clinical results [8]. A number of angiogenic growth factors are in pre-clinical and clinical development for wound healing, including vascular endothelial growth factor (VEGF), fibroblast growth factor (FGF), keratinocyte growth factor (KGF), and transforming growth factor-beta (TGF-β). Although PDGF-BB was the first growth factor to become clinically approved for wound care, it is likely that multiple growth factors will eventually become available and that combination therapy may be beneficial [7].

Dll4 is an endothelium-specific Notch ligand [9]. *Dll4* is largely an arterial-endothelial-specific gene, especially during embryonic development, while in adults its expression is mostly restricted to small arteries and capillary networks [10], [11]. Haploinsufficiency of *Dll4* in mice results in embryonic lethality at approximately E10,5 due to defective vascular development [10]. Notably, *Dll4* and *Vegf* are the only known genes with a haploinsufficient-lethal phenotype due to failure to form a functional vasculature.

Recently, several groups have investigated whether inhibition of the Dll4/Notch pathway might affect tumor angiogenesis and growth. Notch pathway blockade was achieved with either a Dll4-selective neutralizing antibody or a soluble Dll4 fusion protein (sDll4-Fc) that presumably works by binding Notch receptors and preventing their activation by endogenous Dll4 [12], [13], [14]. These biopharmaceuticals showed robust antitumor activity in a variety of human and rodent tumor xenograft models. Histology of sDll4-Fc treated tumors revealed that the reduced tumor growth was associated with an increase in tumor vascular density. However, labeling with an intravascular tracer found these blood vessels to be poorly perfused. Inefficient blood flow in the treated tumor vessels was also reflected by the increased hypoxia observed in tumors treated with sDll4-Fc. It appears that excessive branching results in a highly chaotic vascular network that lacks the hierarchy essential for efficient directional blood flow [12], [13], [14].

Notch signaling impact on wound healing has been previously tested [15], revealing that inhibition of generalized Notch1 signaling leads to delayed wound healing. However the results were not specific for the endothelium because Notch1 is known to be expressed in several cell types involved in skin regeneration, like keratinocytes and immune cells, among others. The endothelial specificity of Dll4 makes it a good potential target for Notch signaling manipulation in the endothelium. Dll4 appears to function as anti-angiogenic factor, negatively regulating pro-angiogenic factors, such as VEGF, and positively regulating vascular maturation factors, like TGF-β [16], [17]. Thus, anti-Dll4 therapy results in increased vascularization with decreased vascular maturation and, therefore, decreased vascular function [18]. The same scenario was observed in the tumor tests [12], [13], [14]. We assumed that as Dll4 function is very dose-dependent, the effect of this therapy might be adjusted by modulating drug dosage. Here we provide *in vivo* evidence supporting the use of anti-Dll4 therapy to increase vascularization without decreasing vascular function, improving the rate of wound healing.

## Methods

### Experimental animals

All animal-involving procedures in this study were approved by the Faculty of Veterinary Medicine of Lisbon Ethics and Animal Welfare Committee (Approval ID: PTDC/CVT/71406/2006). The generation of *Dll4^+/−^* (Dll4/LacZ) mice on CD1 background has been reported previously [10]. *Dll4* conditional knockout mice (*Dll4^lox/lox^*) have been obtained in the lab, in collaboration with Dr. Freddy Radtke [19]. These were crossed with *VE-cadherin-Cre-ERT2* mice, a kind gift by Dr. Ralph Adams, to produce endothelial-specific inducible loss-of-function (*eDll4^lox/lox^*) that is dependent on tamoxifen administration (50 mg/kg daily for 5 days, starting on week 4). Control mice have the same genotype but are uninduced. *Dll4* conditional heterozygote mice are Dll4^+/lox^*VE-cadherin-Cre-ERT2 (*eDll4^+/lox^*) and are induced by tamoxifen. Control mice for both mutants have the same genotype as the respective mutant but are uninduced. Dll4 conditional gain-of-function mice, *tetO7-Dll4* [16], were crossed with Tie2-rtTA mice. Doxycycline (4 mg/ml in drinking water from week 4) was given to double transgenic offspring throughout the experiment, inducing endothelial-specific transgene expression (*eDll4OE*). The control mice have the same *Dll4* gain-of-function (GOF) conditional genotype but were not induced with doxycycline. In the therapeutical trials sDll4-Fc (soluble Dll4 extracellular domain fused to human IgG1 Fc) was systemically administered by intraperitoneal injection every 48 h to C57BL/6 mice from experimental day 0 until endpoint. Control mice were administered an equal volume of PBS. sDll4-Fc was produced as previously described [12].

### Cutaneous wounding procedure

Male mice of 10 to 15 weeks of age were used. A punch wound was created on the back of each mouse as described [15]. Briefly, the hair on the back of the mouse was shaved and two full-thickness wounds were created on each mouse by excising the skin and the underlying *panniculus carnosus* with a 4 mm dermal biopsy punch. Wounds were measured on day 0, to serve as reference, and periodically at each 24 h from that point onwards. Wounds were considered to be ellipsoid in shape and measurements of the larger (L) and smaller (S) diameters of each wound were made with a caliper by two independent investigators. Percentages of the initial wound areas were calculated as: [(π×L×S) on day n/(π×L×S) on day 0×100] and presented as (%). Experimental end-point was determined as the point where the majority of mice had wound sizes below 10% of the original size (<0,4 mm diameter), as at that point measuring error was considered to outweigh measuring accuracy. Since the end-point varied for each genetic background, the choice of intermediate days for analysis was based on two time points per experiment where wound sizes were found to be more different between experimental groups.

### Tissue preparation and immunohistochemistry

On each end-point mice were sacrificed and wounds were recovered. Biopsies were fixed in 4% paraformaldehyde (PFA) solution at 4°C for 1 h, cryoprotected in 15% sucrose, embedded in 7,5% gelatine, frozen in liquid nitrogen and cryosectioned at 20 µm. Double fluorescent immunostaining to platelet endothelial cell adhesion molecule (PECAM) and pericyte marker alpha smooth muscle actin (α-SMA) was performed on tissue sections to examine wound vascular density and vessel maturity. Rat monoclonal anti-mouse PECAM (BD Pharmingen, San Jose, CA) and rabbit polyclonal anti-mouse α-SMA (Abcam, Cambridge, UK) were used as primary antibodies and species-specific conjugated with Alexa Fluor 488 and 555 (Invitrogen, Carlsbad, CA) were engaged as secondary antibodies. Tissue sections were incubated with primary antibody overnight at 4°C and secondary antibody for 1 hour at room temperature. Nuclei were counterstained with 4′, 6-diamidino-2-phenylindole dihydrochloride hydrate (DAPI; Molecular Probes, Eugene, OR). Fluorescent immunostained sections were examined under a Leica DMRA2 fluorescence microscope with Leica HC PL Fluotar 10 and 20X/0,5 NA dry objective, captured using Photometrics CoolSNAP HQ, (Photometrics, Friedland, Denmark), and processed with Metamorph 4.6–5 (Molecular Devices, Sunnyvale, CA). Morphometric analyses were performed using the NIH ImageJ 1.37v program. Vessel density corresponds to the percentage of each tumor section field occupied by a PECAM-positive signal (as determined by the percentage of white pixels per field after transforming the RGB images into binary files). As a measure of vascular maturity, mural cell recruitment was assessed by quantitating the percentage of PECAM-positive structures lined by α-SMA-positive coverage.

### Vascular perfusion and extravasation

To mark vessel perfusion, tribromoethanol anesthetized mice were injected with biotin-conjugated lectin from *Lycopersicon esculentum* (100 µg in 100 µl of PBS; Sigma, St. Luis, MO) via caudal vein and allowed to circulate for 5 minutes before vessel perfusion with 4% PFA in PBS for 3 minutes. Wound samples were collected and processed as presented above. Tissue sections (20 µm) were stained with rat monoclonal anti-mouse PECAM (BD Pharmingen, San Jose, CA), followed by Alexa 555 goat anti-rat IgG (Invitrogen, Carlsbad, CA). Biotinylated lectin was visualised with Streptavidin-Alexa 488 (Invitrogen, Carlsbad, CA). The images were obtained and processed as described above. Wound perfusion was quantified by determining the percentage of PECAM-positive structures that were colocalized with Alexa 488 signals. To analyze vascular extravasation tribromoethanol anesthetized mice were injected with 1% Evans Blue solution (Sigma, St. Luis, MO) via caudal vein and allowed to circulate for 5 minutes before transcardial vessel perfusion with 4% PFA in PBS for 3 minutes. Evans Blue is red fluorescent and extravasation was visualized in contrast to green fluorescent vascular structures and compared between groups [20].

### Quantitative transcriptional analysis

Biopsies were recovered by dissecting wound tissue from experimental mice and snap frozen in liquid nitrogen until RNA extraction (Qiagens RNeasy). Using a SuperScript III FirstStrand Synthesis Supermix qRTPCR (Invitrogen, Carlsbad, CA), first-strand cDNA was synthesized from total RNA. Real-time PCR analysis was performed as described [16], using specific primers. Primer pair sequences are available on request. Gene expression was normalized to β-*actin*.

### Statistical analyses

Data processing was carried out by engaging Statistical Package for the Social Sciences version 17.0 (SPSS v. 17.0; Chicago, IL). Statistical analyses were performed using Mann-Whitney-Wilcoxon test. All results are presented as mean ± SEM. *P*-values<0,05 and <0,01 were considered significant (indicated in the Figures with *) and highly significant (indicated with **), respectively.

## Results

### Dll4 signaling inhibition leads to antagonistic effects on wound healing

**Figure 1:**
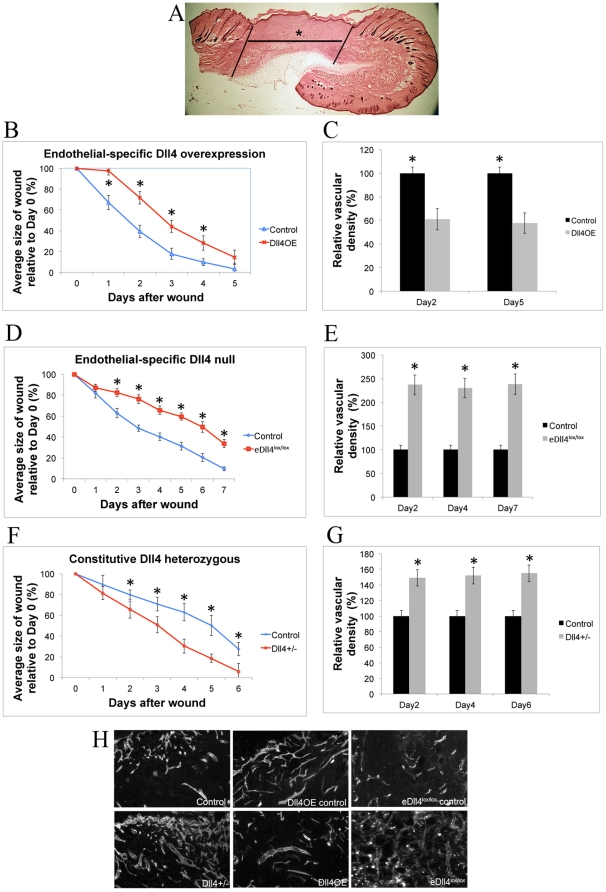
Wound regeneration in *Dll4* mouse mutants. A) Hematoxylin-Eosin staining of a wound biopsy cryosection. Lines delimit the wound margins, (*) denotes granulation tissue. All immunofluorescence images relate to neo-vasculature formed inside granulation tissue. B) Comparison of *eDll4OE* mice with uninduced controls. Graphic depicting the correlation between wound areas in each experimental day relative to the wound area measured on Day 0, Wound regeneration is delayed in *eDll4OE* mice. C) Vascular density is decreased in granulation tissue of *eDll4OE* mice, relative to uninduced controls throughout the experiment. D) Comparison of *eDll4^lox/lox^* mice with uninduced controls. Graphic depicting the correlation between wound areas in experimental days relative to wound areas measured on Day 0, Wound regeneration is delayed in *eDll4^lox/lox^* mice. E) Vascular density is increased in granulation tissue of *eDll4^lox/lox^* mice, relative to uninduced controls throughout the experiment. F) Graphic depicting the correlation between wound areas in experimental days relative to wound areas measured on Day 0, comparing *Dll4^+/−^* mice with wild type (WT) controls. Wound regeneration is accelerated in *Dll4^+/−^* mice. G) Vascular density is increased in granulation tissue of *Dll4^+/−^* mice, relative to WT controls throughout the experiment. H) Representative anti-PECAM immunofluorescence images of neo-vasculature in granulation tissue of above described genetic models at endpoint. * In graphics represents p<0,05.

The study of the impact of Dll4 signal modulation was initially carried out using genetic mouse models of *Dll4* loss- and gain-of-function. Wound healing assays on an endothelial-specific *Dll4* overexpressing mouse line (*eDll4OE*) and an endothelial-specific *Dll4* conditional *knockout* mouse line (*eDll4^lox/lox^*) were anticipated to provide some insight on the role of Dll4 in regulating adult neo-angiogenesis. We performed daily wound area measurements and evaluated vascular density in the granulation tissue of the wound (Fig. 1A). Wound healing was impaired in both eDll4OE and eDll4^lox/lox^ mice (Fig. 1B, D). Significantly delayed wound healing was observed in *eDll4OE* mice as early as day 1 and was maintained until day 4, when wounds were reduced to around 10% of their original size. Over a 5-day period, there was a 1 to 1,5 days delay in wound healing in *eDll4OE* mice compared to controls. Histological analysis on days 2 and 5 revealed that *eDll4OE* mice had approximately 40% lower vascular density in the wound region than controls (Fig. 1C). Absolute vascular density values increased over the period of regeneration in both groups but the difference between groups was maintained or slightly increased (data not shown). Wound healing was also delayed in *eDll4^lox/lox^* mice (Fig. 1D), which was statistically significant at day 2 and persisted. Overall there was a 2 to 3 days delay compared to control group over a 7-day period. Histological analysis on days 2, 4 and 7 revealed a large increase in vascular density throughout the experimental period in *eDll4^lox/lox^* mice (Fig. 1E), consistent with the previous report that Dll4 loss promotes angiogenesis [12], [21].

In contrast to the initial findings, wound-healing assays on *Dll4^+/−^* mice revealed that regeneration was improved when compared with control *Dll4^+/+^* mice (Fig. 1F). Differences in wound size became statistically significant by day 2 and persisted until day 6. From day 2 until day 6 the wound healing in *Dll4^+/−^* mice presented a steady 2-day advance over control mice. Histological analysis revealed an increase in vascular density that could be seen in *Dll4^+/−^* mice as early as day 2 (Fig. 1G). This difference in vascular density was maintained throughout the wound recovery period, even though absolute vascular density values increase in both experimental groups (data not shown).

**Figure 2:**
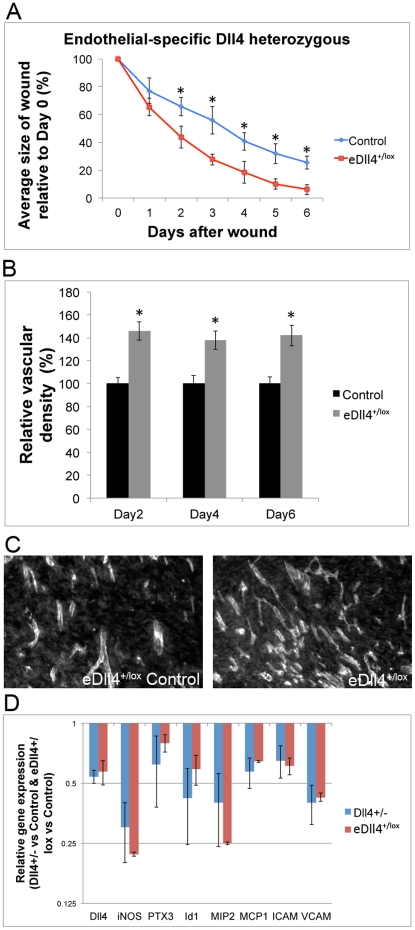
The wound regeneration profile of endothelial specific Dll4 heterozygous mice (*eDll4^+/lox^*) is similar to that of constitutive Dll4 heterozygous mice (*Dll4^+/−^*) and both share a reduced inflammatory response when compared to *Dll4* WT mice. A) Comparison of *eDll4^+/lox^* mice with uninduced controls. Graphic depicting the correlation between wound areas in each experimental day relative to the wound area measured on Day 0, Wound regeneration is accelerated in *eDll4^+/lox^* mice in a similar way as in the *Dll4^+/−^* mice. B) Vascular density is increased in granulation tissue of *eDll4^+/lox^* mice, relative to uninduced controls throughout the experiment. C) Representative anti-PECAM immunofluorescence images of neo-vasculature in granulation tissue of *eDll4^+/lox^* and uninduced controls at endpoint. D) Differential gene expression in *Dll4^+/−^* versus WT and *eDll4^+/lox^* versus uninduced control wounds of inflammation-related genes at day 2. *Dll4* expression is confirmed to be approximately 0,5-fold in both cases, relative to their respective controls, and statistically similar. Inflammation-related genes have reduced expression in both mutant mice, probably resulting from the improved condition of the mutant mice wounds that regenerate faster. * In graphics represents p<0,05.

To confirm that the improvement in wound healing observed in *Dll4^+/−^* mice was due to the change in vasculature, and not others factors such as the inflammatory response [22], further experiments with endothelial-specific conditional *Dll4* heterozygotes (Dll4^+/lox^*VE-Cadherin-CreERT2 - from now on *eDll4^+/lox^*) were carried out. Results revealed that in *eDll4^+/lox^* mice wounds regenerated faster than in the respective controls, in a similar fashion to what was described for *Dll4^+/−^* mice. A two-day advance in wound closure of *eDll4^+/lox^* was established by day 2 and was maintained until the endpoint (Fig. 2A). Vascular density evaluation confirmed identical differences in relation to wild type controls in both *eDll4^+/lox^* and *Dll4^+/−^* (Fig. 2B,C). Pro-inflammatory gene expression at day 2, during the inflammatory phase of wound closure, was tested in both *Dll4^+/−^* and *eDll4^+/lox^* mice. This would allow the identification of a possible influence of Dll4 function in mediating the inflammatory response independent of the vascular phenotype. This possible effect was tested by RT-PCR analysis of wound biopsies to evaluate the expression of pro-inflammatory genes. Results showed that monocyte/macrophage chemo attractant MCP1 had reduced expression in both *eDll4^+/lox^* and *Dll4^+/−^*. Pro-inflammatory genes, such as ICAM, VCAM and MIP2, were also downregulated in both *eDll4^+/lox^* and *Dll4^+/−^* mice. Markers of macrophage activation iNOS, PTX3 and Id1 had reduced expression in both *eDll4^+/lox^* and in *Dll4^+/−^* (Fig. 2D). We then evaluated the gene expression profile of the same inflammation-related genes in the *Dll4* mutant mice that showed impaired regeneration profile, *eDll4^lox/lox^* and *Dll4OE*, as this would allow us to correlate changes in the inflammatory profile of wounds to their regeneration profile. Results showed that the expression of pro-inflammatory genes was upregulated in both *eDll4^lox/lox^* and *Dll4OE* (Fig. S1).

### sDll4-Fc therapy has antagonistic effects on wound healing depending on dosage

**Figure 3:**
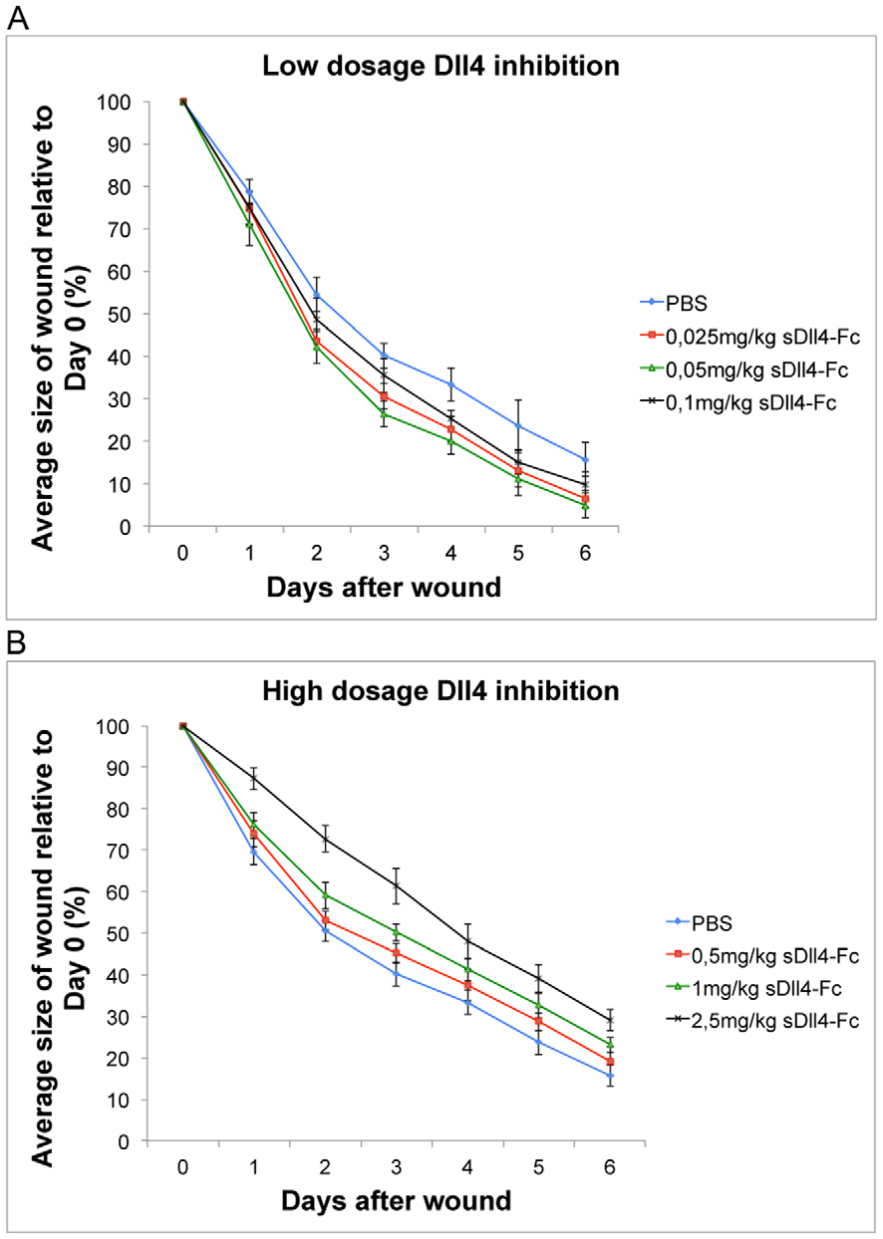
Modulation of sDll4-Fc dosage can achieve both improved or impaired wound regeneration through low- or high-dosage inhibition. A) Graphic depicting the correlation between wound areas in experimental days relative to wound areas measured on Day 0, comparing mice treated with sDll4-Fc dosages lower than 0,1 mg/kg with untreated (sham treated) controls. Wound regeneration is accelerated in mice treated with sDll4-Fc dosages lower than 0,1 mg/kg. B) Graphic depicting the correlation between wound areas in experimental days relative to wound areas measured on Day 0, comparing mice treated with sDll4-Fc dosages higher than 0,5 mg/kg with controls. Wound regeneration is delayed in mice treated with sDll4-Fc dosages higher than 0,5 mg/kg.

**Figure 4:**
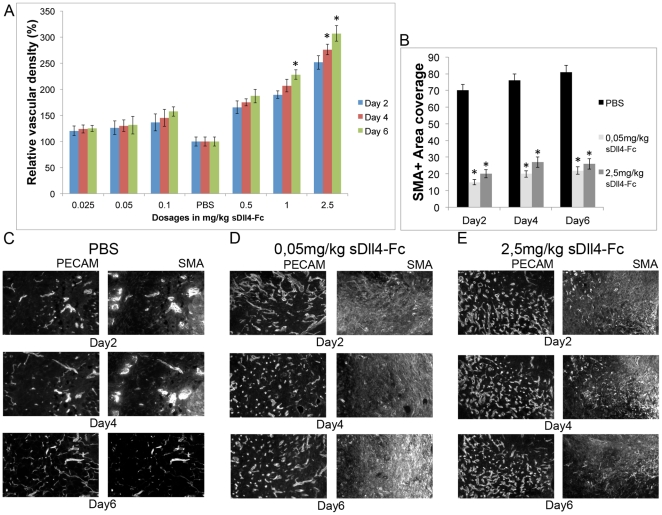
Wounds treated with sDll4-Fc have increased vascular density but decreased vascular maturation in direct proportion to the dosage level. A) Vascular density is increased in granulation tissue of mice treated with sDll4-Fc, being directly proportional to the dosage increase, when compared to controls throughout the experiment. B) Vascular smooth muscle cell coverage is decreased about 3-fold in relation to PBS-injected control mice throughout the experimental days in both tested groups. C,D,E) Representative anti-PECAM and anti-SMA immunofluorescence images of neo-vasculature in granulation tissue of wounds treated with (D) 0,05 mg/kg or (E) 2,5 mg/kg compared with control mice injected with PBS (C), in days 2, 4 and 6. sDll4-Fc therapy leads to decreased smooth muscle cell recruitment and increased vascular density that is directly proportional to the administered dosage. * In graphics represents p<0,05.

Inflammatory gene expression in *eDll4^+/lox^* indicated that Dll4 function in the endothelium was the most important factor accounting for the observed improvements in wound closure. *eDll4^+/lox^* (and *Dll4^+/−^*) and *eDll4^lox/lox^* mice can give rise to opposing phenotypes despite both being loss-of-function mutants and both displaying a pro-angiogenic phenotype. We therefore proposed that dosage of the inhibitor (soluble Dll4-Fc) may mimic the Dll4 dose response observed in *Dll4* deficient mouse lines allowing us to define the dosage of sDll4-Fc that promotes wound healing. sDll4-Fc therapy was tested in C57BL/6 mice using dosages from 0,025 mg/kg to 2,5 mg/kg. Mice were injected on day 0, after wounding, and every 2 days until the endpoint. Lower dosages, like 0,025 mg/kg, 0,05 mg/kg and 0,1 mg/kg, were observed to accelerate wound healing (Fig. 3A). Statistical significance in wound size difference was achieved as early as day 1 in the 0,05 mg/kg dosage group and day 2 in the 0,025 mg/kg and 0,1 mg/kg dosage groups and was maintained throughout the regeneration period. From day 2 until day 5 a 1 to 1,5 day advantage in wound size was observed in treated mice over control mice, with the 0,05 mg/kg dosage group displaying the largest benefit. Testing with higher dosages, such as 0,5 mg/kg, 1 mg/kg and 2,5 mg/kg revealed an opposite effect delaying wound healing (Fig. 3B). Statistical significance in wound size difference was observed by day 1 in the 1 mg/kg and 2,5 mg/kg dosage groups and maintained until endpoint. Dosage 0,5 mg/kg was considered to originate wound healing statistically indistinguishable from that of control mice. Dosage 1 mg/kg displayed a 0,5 to 1 day delay in wound healing, while the 2.5 mg/kg dosage displayed a 1 to 1,5 day delay, over a 6-day period. To understand how opposing effects on wound regeneration were generated by modulating sDll4-Fc therapy dosage we analyzed vascular density on days 2, 4 and 6. This revealed that in all dosage groups there was increased vascular density, the degree of which was in direct relationship with the increase in dosage (Fig. 4 A,C,D,E). Dosages that led to improved wound healing caused increases in vascular density of 20 to 50%, while dosages that led to impaired wound healing caused much larger increases of 70 to 300%.

### Neo-vasculature in sDll4-Fc treated mice is less mature but has variable perfusion and extravasation depending on dosage

Normal neo-vascularization involves the proliferation and progression of pre-existing endothelium into stressed areas. That newly formed vasculature has to mature, becoming more stable. This is achieved mainly by inducing the differentiation and recruitment of smooth muscle cells and pericytes. We analyzed the wound basin neo-vasculature of mice treated with 0,05 mg/kg and 2,5 mg/kg of sDll4-Fc, the dosages that led to the best and worst result in wound healing, and the respective control, for the presence of vascular smooth muscle cells. While PBS-injected mice had a normal coverage of smooth muscle, sDll4-Fc injected mice had a 3-fold reduced coverage in both doses (Fig. 4B, C, D and E). This effect became evident as early as Day 2 and persisted throughout wound regeneration. By comparison, all *Dll4* loss-of-function mutants analyzed in this work produced similar results to those obtained with sDll4-Fc showing reduced presence of perivascular cells, while *Dll4OE* mutants displayed increased recruitment of perivascular cells (Fig. S2).

**Figure 5:**
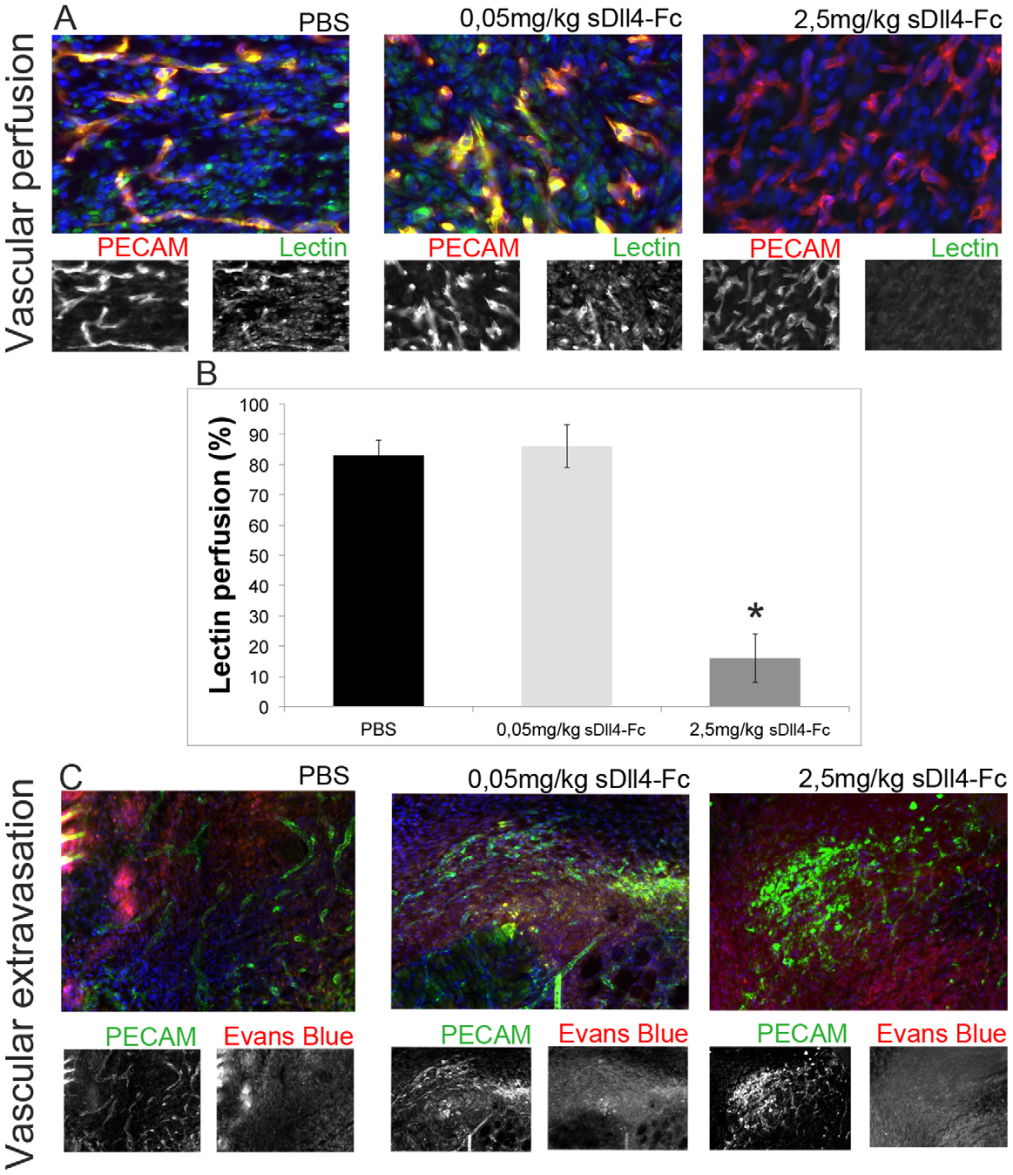
Low-dosage sDll4-Fc does not affect perfusion levels and leads to only a small increase in extravasation while higher dosages cause decreased perfusion and highly increased extravasation. A) Representative anti-PECAM and anti-Lectin immunofluorescence images of neo-vasculature in granulation tissue of wounds treated with 0,05 mg/kg or 2,5 mg/kg compared with control mice injected with PBS, in day 6. B) The percentage of lectin-perfused blood vessels is similar between PBS- and 0,05 mg/kg sDll4-Fc injected mice but since vascular density is increased in the latter this result represents an effective increase in vascular function in the wound basin. Vascular perfusion is highly decreased in wounds of mice injected with 2,5 mg/kg sDll4-Fc. C) Representative anti-PECAM and Evans Blue immunofluorescence images of neo-vasculature in granulation tissue of wounds treated with 0,05 mg/kg or 2,5 mg/kg compared with control mice injected with PBS, in day 6. Vascular extravasation is highly increased in wounds of mice injected with 2,5 mg/kg sDll4-Fc but displays only a small increase in the case of 0,05 mg/kg sDll4-Fc injected mice. * In graphics represents p<0,05.

Since one of the hallmark observations after sDll4-Fc therapy on tumors was the poor perfusion of newly formed vasculature [12], lectin perfusion (Fig. 5A,B) and Evans Blue vascular extravasation (Fig. 5C) testing were carried out in the wound healing setting. The 0,05 mg/kg dosage revealed no significant improvement in the percentage of lectin perfused blood vessels, and a low level of vascular extravasation, relative to control. The 2.5 mg/kg dosage revealed much-decreased lectin perfusion and very pronounced Evans Blue extravasation. These results confirm that the impaired maturation of the neo-vasculature in sDll4-Fc treated mice is dosage-dependent and that the effectiveness of blood circulation to affected tissues varies directly with the degree of neo-vasculature maturation. For comparison purposes, *Dll4^+/−^* and *eDll4^+/lox^* neovasculature had a lectin perfusion index that was not statistically different to their respective controls, and slightly higher Evans Blue extravasation than controls. *Dll4* mutants with impaired wound healing profile, *eDll4^lox/lox^* and *Dll4OE,* both revealed reduced lectin perfusion, while *eDll4^lox/lox^* displayed a very high level of vascular extravasation and *Dll4O*E showed an almost normal level of vascular extravasation (Fig. S3A, B).

### Dll4 modulation has no effect on the skin adjacent to the wound

**Figure 6:**
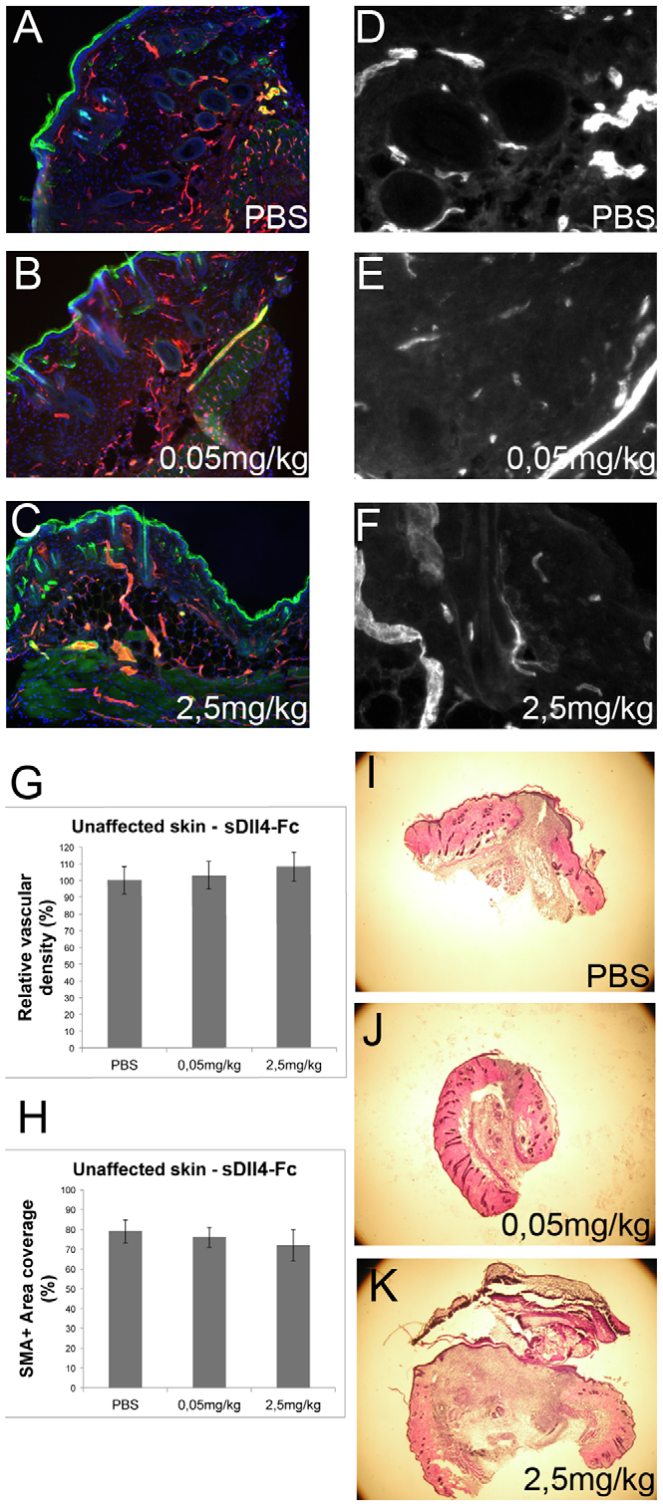
Skin adjacent to the wound site suffers no alterations independently of the dosage used in the sDll4-Fc therapy. Anti-PECAM (red) and anti-SMA (green) immunofluorescence of A) PBS-, B) 0,05 mg/kg sDll4-Fc- or C) 2,5 mg/kg sDll4-Fc-treated mice D,E,F) Representative anti-PECAM immunofluorescence images of neo-vasculature in unaffected skin adjacent to the wound site. G) Vascular density in unaffected skin is not statistically different between PBS-, 0,05 mg/kg- and 2,5 mg/kg-treated mice. The same happens for perivascular cell coverage H). I–J) Hematoxylin and eosin staining of 20 µm cryosections of PBS-, 0,05 mg/kg- and 2,5 mg/kg-treated mice wounds depicting the wound size and unaffected adjacent skin at day 6.

We have described that sDll4-Fc therapy can affect the vasculature of wounds and therefore, influence the regenerative profile. Since our approach is not targeted to the affected region we decided to investigate if the vasculature of skin adjacent to the wound site would be equally affected by sDll4-Fc therapy in either high- or low-dosage form. PECAM and SMA immunofluorescence (Fig. 6A–F) revealed that outside the wound the vasculature was largely unaffected during the therapy period. Vascular density and smooth muscle cell coverage indexes of the skin adjacent to the wound in mice treated with 0,05 mg/kg and 2,5 mg/kg sDll4-Fc did not differ statistically at endpoint from PBS-treated mice (Fig. 6G,H). Microscopic analysis of surrounding tissues also did not reveal any histological changes to control (Fig. 6I–K).

### Effect of Dll4 modulation on the expression of vascular genes

**Figure 7:**
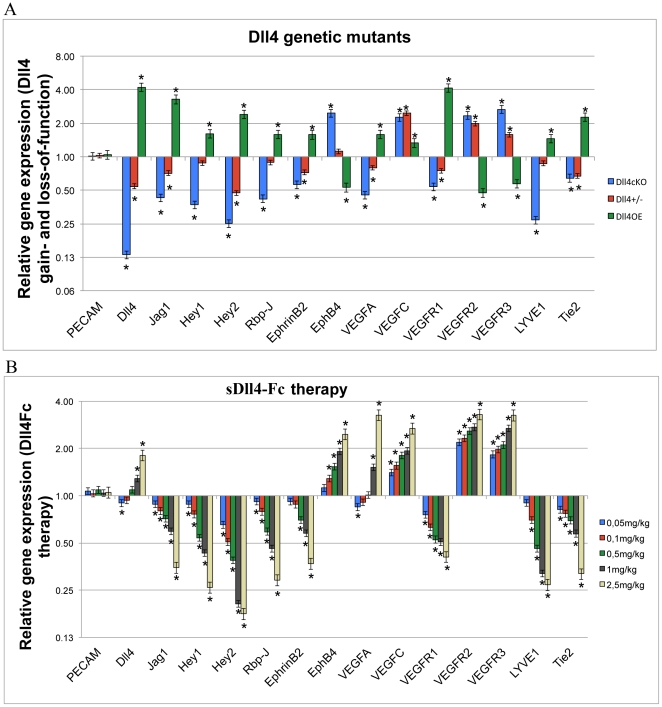
Differential gene expression in wounds affected by A) Dll4 genetic alterations or B) sDll4-Fc therapy administration. Gene expression analysis of wound tissues was performed by RT-PCR for indicated genes involved in angiogenesis. Gene expression levels were normalized for *β-actin* levels. Error bars represent SD. * in graphics represents p<0,05.

To help the identification of the mechanistic causes behind the observed phenotypes the expression of selected genes in the wound area was characterized by RT-PCR. RNA was extracted from wound biopsies taken from both genetic models and sDll4-Fc treated mice. Expression levels were normalized to *PECAM-1* mRNA levels [23] to compensate for variations in vascular density between samples. In the genetic models (Fig. 7A), *Dll4* expression levels corresponded to the expectation, with a 4-fold increase in the *Dll4OE* mice and a reduction to 0,5× in the *Dll4^+/−^* and to 0,13× in the *eDll4^lox/lox^* mice. *Jagged1* expression levels decreased in the loss-of-function models and increased in the *Dll4OE* mice. *Hey2*, the main known Notch signaling effector in the endothelium [24], displayed higher expression levels in *eDll4OE* and lower expression levels in the loss-of-function models, confirming that the Notch pathway is being affected by the induced *Dll4* mutations. *Hey1* gene expression levels were found to vary similarly to *Hey2* except that the differences to respective controls were smaller. *Rbp-J* is the principal mediator of NICD function in the regulation of Notch effector genes expression regulation [25]. *Rbp-J* expression levels were found to be upregulated in *eDll4OE* and downregulated in the Dll4 loss-of-function models. Ephrin-B2 is known to be downstream of Notch signaling in embryonic vascular development and is a regulator of vascular maturation and endothelial identity [26]. *Ephrin-B2* expression was decreased in *Dll4^+/−^* and *eDll4^lox/lox^* mice and increased in *eDll4OE* mice, corroborating the vascular maturation results obtained by immunofluorescence. *EphB4* encodes the venous-specific cognate receptor for Ephrin-B2 [27]. *EphB4* expression was upregulated in *eDll4^lox/lox^* and downregulated in *Dll4OE* mice. Dll4 is a known regulator of VEGF signaling, having specific control over the expression of its transmembrane receptors in the endothelium [28]. *Vegf-a* expression levels were decreased in *Dll4^+/−^ mice*, while they were increased in the *eDll4^lox/lox^* and *eDll4OE* mice, which had delayed wound regeneration, probably accounting for reduced hypoxia in wounds with improved regeneration. Dll4 is a known suppressor of VEGF-C/VEGFR3 signaling [29]. The expression of *Vegf-c* in the wounds was augmented in both *Dll4^+/−^* and *eDll4^lox/lox^* mice but in *eDll4OE* mice the difference was found to be not significant. VEGF receptor expression analysis revealed that *eDll4OE* mice had lower expression of *Vegfr2* and *Vegfr3*, both of which mediate VEGF signaling, and higher expression of *Vegfr1*, which acts mainly as a VEGF trap [30] and blocks VEGF-A mediated pro-angiogenic signal. In the loss-of-function models VEGFRs expression varied in the opposite direction, with decreased *Vegfr1* expression and increased *Vegfr2* and *Vegfr3* expression and thus increased angiogenic potential. Tie2 loss-of-function is associated with a decrease in smooth muscle cell recruitment [31]. Expression of *Tie2* in our genetic models reflects this effect, being lower in both *Dll4^+/−^* and *eDll4^lox/lox^* mice, which had less smooth muscle, and increased in *eDll4OE* mice, which had an higher than normal number of smooth muscle cells. *Lyve1* is a known lymphatic endothelial cell-specific gene [32]. Recently, Dll4 blockade was associated with disruption of post-natal lymphatic development [33]. *Lyve1* expression was downregulated in *eDll4^lox/lox^* mice and upregulated in *eDll4OE*. More importantly *Lyve1* expression in *eDll4^+/lox^*, which displayed accelerated wound healing, was found to be near normal levels.

In the mice subjected to sDll4-Fc therapy (Fig. 7B), endogenous *Dll4* expression decreased with dosages up to 0,1 mg/kg and increased with dosages greater than 0,5 mg/kg, reflecting *Vegf-a* expression levels, one of the main inducers of *Dll4* expression. *Hey2* expression levels confirmed the inhibitory effect of sDll4-Fc on Notch signaling, being lower in all dosages and showing a strong dependency on the dosage of the inhibitor, again confirming that higher dosages of sDll4-Fc lead to increasing inhibition of Dll4/Notch signaling. *Jagged1*, *Hey1 and Rbp-J* expression values were similar to that of *Hey2*. *Ephrin-B2* expression also responded linearly to increasing sDll4-Fc inhibition, showing greater decrease with increasing sDll4-Fc dosage. In contrast *EphB4* expression increased in direct relation to sDll4-Fc dosage. *Vegf-c* expression also increased in direct relation to sDll4-Fc dosage. In regard to the VEGF receptors, expression of *Vegfr1* decreased (in indirect relation to sDll4-Fc dosage) and *Vegfr2* and *Vegfr3* increased in direct relation to sDll4-Fc dosages. *Tie2* expression levels decreased in all sDll4-Fc dosages, showing an inverse relation to sDll4-Fc dosages. *Lyve1* expression values were always downregulated, except at the 0,05 mg/kg sDll4-Fc dosage, with expression decreasing with increasing sDll4-Fc dosages.

## Discussion

The importance of Dll4 was first described by its function in the establishment of endothelial identity and in the regulation of vascular morphogenesis in embryonic vascular development. Experimental data place Dll4 in the middle of a loop of angiogenesis regulatory pathways. Dll4 was found to be one of the key players acting as negative regulator of angiogenesis, working to maintain the fine balance between vascular progression and vascular maturation/quiescence [34]. This important function has led to the development of therapies blocking Dll4 function for tumor therapy. Dll4 inhibition, and sDll4-Fc therapy in particular, have shown potential in controlling tumor growth, even in cases when tumors have gained resistance to anti-VEGF therapies [12], [13]. The mechanism of action of this therapy is strikingly different to that of classic anti-angiogenic therapies, which work by reducing the ability of a tumor to induce the growth of new blood vessels or by reducing the ability of blood vessels to sprout and form the neo-vasculature in the tumor. Dll4 inhibition works by increasing vascular density through uncontrolled growth, resulting in the new vasculature being disorganized, inefficient and poorly perfused, leading to an increase in tumor hypoxia and reduction in tumor growth [35].

As the embryonic lethal haploinsufficiency of *Dll4* is indicative of a strong functional dosage-dependence, we hypothesized that the non-functional nature of the tumoral neo-vasculature observed in anti-Dll4 therapy [12], [21] was a consequence of the high dosage of Dll4 inhibitor used and that by modulating therapy dosage it might be possible to achieve a functional pro-angiogenic effect. Dll4-based therapy could then be used to treat conditions where the formation of new blood vessels is essential and beneficial, like wound healing. In this case, angiogenesis starts almost immediately following injury and is important for the formation of granulation tissue, to supply oxygen and nutrients to growing tissues and as a means to deliver inflammatory cells to the wound site [4].

Wound healing was tested on murine genetic models of *Dll4* gain- and loss-of-function. Results revealed that Dll4 heterozygote mice (both *Dll4^+/−^* and *eDll4^+/lox^*) presented accelerated wound regeneration with improved vascular density as well as near-normal percentage of perfused blood vessels, whereas *eDll4^lox/lox^* and *eDll4OE* presented delayed wound healing. In the first case it was related to highly increased vascular density and in the latter related to a reduction in vascular density, both phenotypes leading to reduced tissue perfusion. Since improved wound regeneration observed in the *Dll4^+/−^* mice was caused by a constitutive mutation and previous works described a possible link between Dll4 and inflammatory macrophages [22], we decided to test if an equivalent endothelial-specific mutation could give rise to the same phenotype. Results with *eDll4^+/lox^* mice showed a similar wound regeneration profile to that of *Dll4^+/−^* mice. This indicates that at least the principal contributor to the observed phenotype was the loss of Dll4 function in the endothelium. Nevertheless we measured by RT-PCR the expression levels of several regulators of inflammatory function. The expression profile indicated that in both *Dll4^+/−^* and *eDll4^+/lox^* there is a reduction in the expression of macrophage chemoattractants, regulators of monocyte/macrophage infiltration and markers of macrophage activation. Since the results are not statistically different between *eDll4^+/lox^* and *Dll4^+/−^* we can infer that the differences in the inflammatory profile of both mutant mouse lines is unrelated to Dll4 expression. The gene expression levels of the same inflammatory profile markers are increased in both the *eDll4^lox/lox^* and *Dll4OE* mice with opposing *Dll4* mutations and delayed wound regeneration. So, the observed inflammatory profiles are likely to be a consequence of an improved or impaired wound regeneration status, not a specific response to the *Dll4* mutations. These results confirmed that different levels of Dll4 blockade give rise to either an improvement or impairment in wound regeneration, depending on whether the increased angiogenesis leads to functional or non-functional blood vessels.

The next step was to test different dosages of sDll4-Fc therapy in surgically wounded wild-type mice. Results showed that lower dosages, between 0,025 mg/kg and 0,1 mg/kg accelerate wound regeneration by inducing productive angiogenesis that is functional and causes an improvement in blood flow in the wound area without affecting the surrounding quiescent vasculature. Comparing results from different dosages, 0,025 mg/kg, 0,05 mg/kg and 0,1 mg/kg revealed that while all dosages tested gradually improved vascular density, the transition from functional to non-functional angiogenesis appears to occur when the dosage increases from 0,05 to 0,1 mg/kg. This is based on the observation that wound healing is accelerated when the dosage increases from 0,025 to 0,05 mg/kg, as a result of increased vascular function, but slowed down when it is further increased from 0,05 to 0,1 mg/kg. This indicates that the transition from functional to non-functional angiogenesis is a result of vascular inefficiency arising from increasing vascular density and inability to form proper blood vessel lumens and perivascular wall. With higher dosages, ranging from 0,5 to 2,5 mg/kg of sDll4-Fc, wound regeneration was progressively delayed over control mice, as increasingly higher vascular densities led to decreased vascular maturation, decreased perfusion and increased vascular extravasation and therefore reduced vascular function. Most importantly, in sDll4-Fc treated mice the vasculature of the skin adjacent to the wound site remained unaffected when compared to PBS-treated mice. This result indicates that sDll4-Fc therapy specifically targets sites of active angiogenesis, where Dll4 function is most important to regulate vascular morphogenesis. A recent work [36] described that prolonged (over 8 weeks long) therapy with high dosages of Dll4 inhibitors (much higher than any of the dosages used in this work) can lead to low frequency non-lethal subcutaneous vascular neoplasms and histopathological changes in the liver. We have not observed subcutaneous tumors in our sDll4-Fc treated mice or *Dll4* mutant mice for the duration of the experiments. It is possible that the lesions cited are also dosage-dependent or due to long term Dll4 blockade. Our results indicate that at low dosages and for short term therapy the risk of such secondary effects should be minimal.

Gene expression analysis confirmed the existence in the wound healing process of a regulatory loop between VEGF signaling and Dll4/Notch signaling, where Dll4/Notch signaling blockade leads to a reduction in *Vegfr1* and an increase in *Vegfr2* and *Vegfr3* expression, increasing vascular response to VEGF-A and –C expression from surrounding stroma [29], [37]. Jagged1 is a member of the Notch ligand family and is present in arterial endothelial cells and vascular smooth muscle cells [39]. *Jagged1* expression was found to be directly related to Dll4 function, which could reveal a novel regulatory feedback loop between Dll4 and Jagged1 where increasing Dll4 function potentiates *Jagged1* expression that then negatively regulates Dll4/Notch signaling [24]. Ephrin-B2 is involved in the activation of angiogenesis and recruitment of perivascular cells to neo-vasculature [38], [39], acting downstream of VEGF and Notch signaling while at the same time acting as a regulator of VEGF-R2 and –R3 internalization and function [40], [41]. It is expressed in arterial endothelium and in smooth muscle cells. *Ephrin-B2* expression was found to be directly related to Dll4 function and could indicate one way through which Dll4/Notch signaling regulates vascular smooth muscle cell recruitment. *EphB4* expression is opposite to *Ephrin-B2* expression and increases with sDll4-Fc dosage. This could also indicate that higher dosages of sDll4-Fc lead to a shift to venous neo-vasculature, while lower dosages have no impact on arterial-venous proportion. The Tie2 receptor is considered to be endothelial-specific, while its cognate ligand Ang1 is expressed mainly by perivascular and mural cells, acting as a paracrine signal to the endothelium. Loss-of-function studies revealed a phenotype with defective angiogenesis and reduced coverage and detachment of smooth muscle cells and pericytes [42]. sDll4-Fc therapy leads to a dosage-dependent reduction in Tie2 expression, demonstrating the Ang1/Tie2 pathway involvement in the regulation of vascular maturation and quiescence downstream of Dll4/Notch signaling. It was shown recently that Dll4 blockade could inhibit lymphatic vessel assembly [33]. Our expression results revealed that while that is true for high dosage Dll4 inhibition, in the 0,05 mg/kg sDll4-Fc dosage the reduction in *Lyve1* expression, a lymphatic endothelial cell marker is not significantly different to the control.

A role for Notch signaling in wound healing [15] has previously been suggested by use of a Notch1 knockdown mouse line as well as small molecule inhibitor of Notch signaling (GSI treatment). Those results indicated that Notch1 inhibition led to a delay in wound regeneration but failed to report on the wound vascular phenotype. Besides endothelial cells, Notch1 is present in skin, at least in keratinocytes as well as in various immune cells, making it difficult to separate the impact of the mutation in the different cell systems and how they integrate to display a given phenotype.

Together, our results present evidence that endothelial Notch signaling is a potential target for wound regeneration therapies through its ligand Dll4. Low-dosage sDll4-Fc accelerates wound regeneration by creating a neo-vasculature that has a slight increase in vascular density while keeping nearly normal perfusion of the blood vessels and arterial-venous phenotype, without affecting lymphatic vasculature. Overall low-dosage sDll4-Fc therapy leads to an effective increase in local blood supply to the wound site, not affecting lymphatic drainage or the quiescent vasculature.

## Supporting Information

Figure S1 *Dll4* mutants with impaired wound regeneration share a pro-inflammatory profile. Differential gene expression in *Dll4OE* and *eDll4^lox/lox^* versus respective uninduced control wounds of inflammation-related genes at day 2. Inflammation-related genes have upregulated expression in both mutant mice, probably resulting from the impaired condition of the mutant mice wounds that regenerate slower. * In graphics represents p<0,05.
(TIF)

Figure S2 All *Dll4* loss-of-function mutants tested displayed reduced perivascular cell recruitment, while *Dll4OE* mice revealed an opposite phenotype. * In graphics represents p<0,05.
(TIF)

Figure S3 A) The percentage of lectin-perfused blood vessels is not statistically different to control levels in *Dll4^+/−^* and *eDll4^+/lox^*, while being highly reduced in *eDll4^lox/lox^* and *Dll4OE*. B) Evans Blue extravasation is slightly increased in *Dll4* heterozygote models, highly increased in *eDll4^lox/lox^* and near normal levels in *Dll4OE*. * In graphics represents p<0,05.
(TIF)
