# Supplementary figures and images for: Low-Dosage Inhibition of Dll4 Signaling Promotes Wound Healing by Inducing Functional Neo-Angiogenesis
Source: PLoS One. 2012 Jan 18;7(1):e29863. doi: 10.1371/journal.pone.0029863 (PMC3261161; doi:10.1371/journal.pone.0029863)

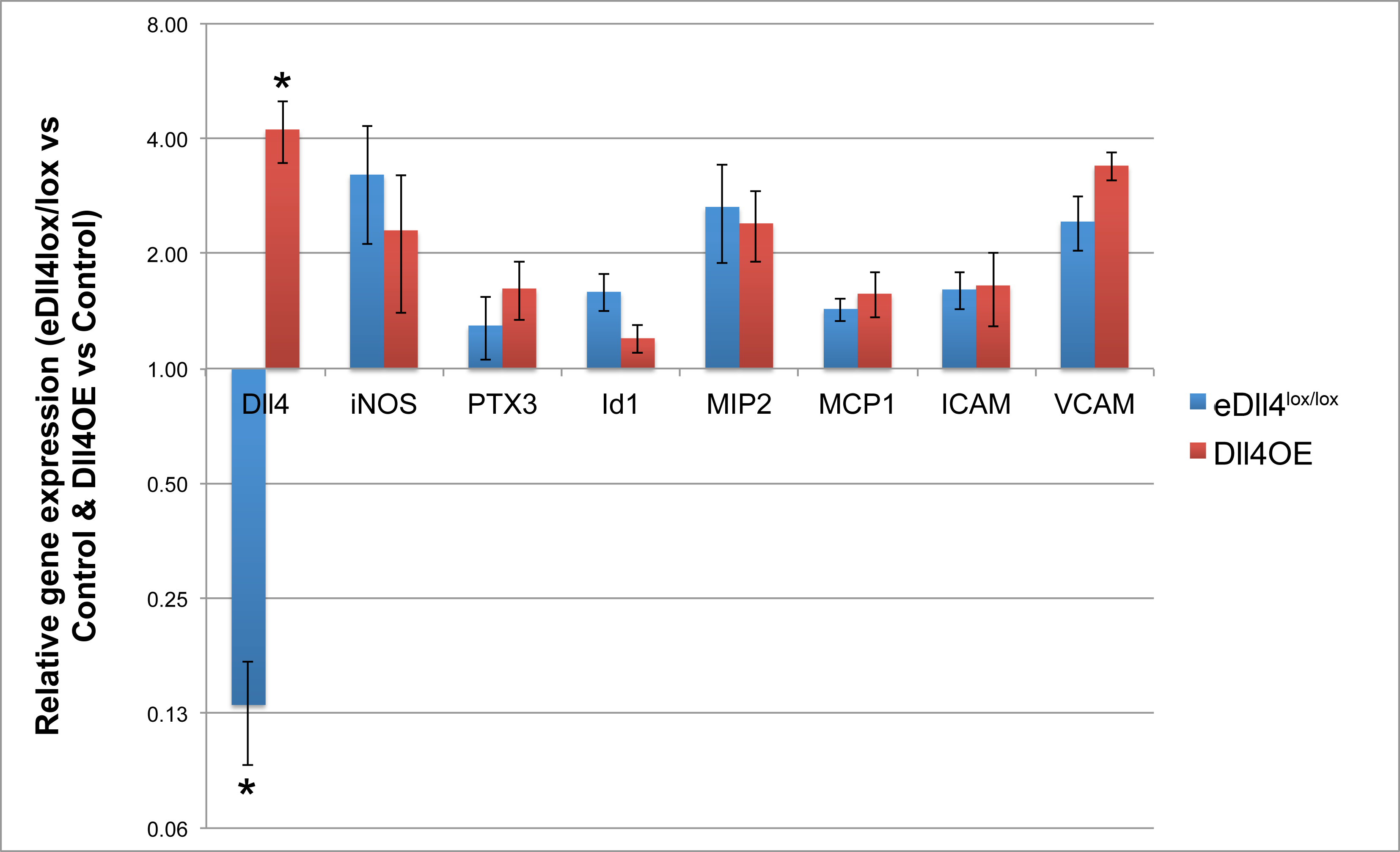

Supplement: Figure S1 — Dll4 mutants with impaired wound regeneration share a pro-inflammatory profile. Differential gene expression in Dll4OE and eDll4lox/lox versus respective uninduced control wounds of inflammation-related genes at day 2. Inflammation-related genes have upregulated expression in both mutant mice, probably resulting from the impaired condition of the mutant mice wounds that regenerate slower. * In graphics represents p<0,05. (TIF) [file pone.0029863.s001.tif]

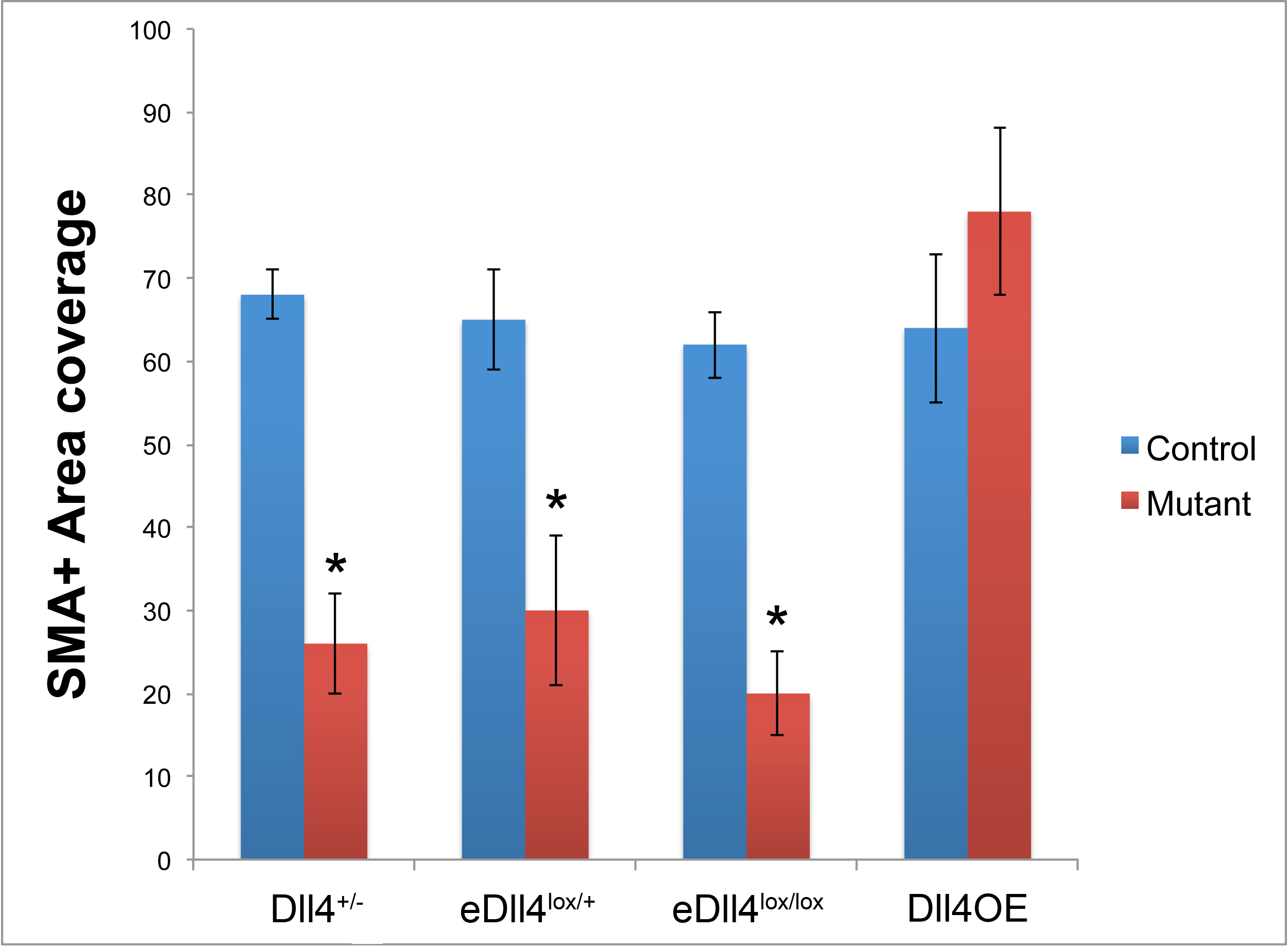

Supplement: Figure S2 — All Dll4 loss-of-function mutants tested displayed reduced perivascular cell recruitment, while Dll4OE mice revealed an opposite phenotype. * In graphics represents p<0,05. (TIF) [file pone.0029863.s002.tif]

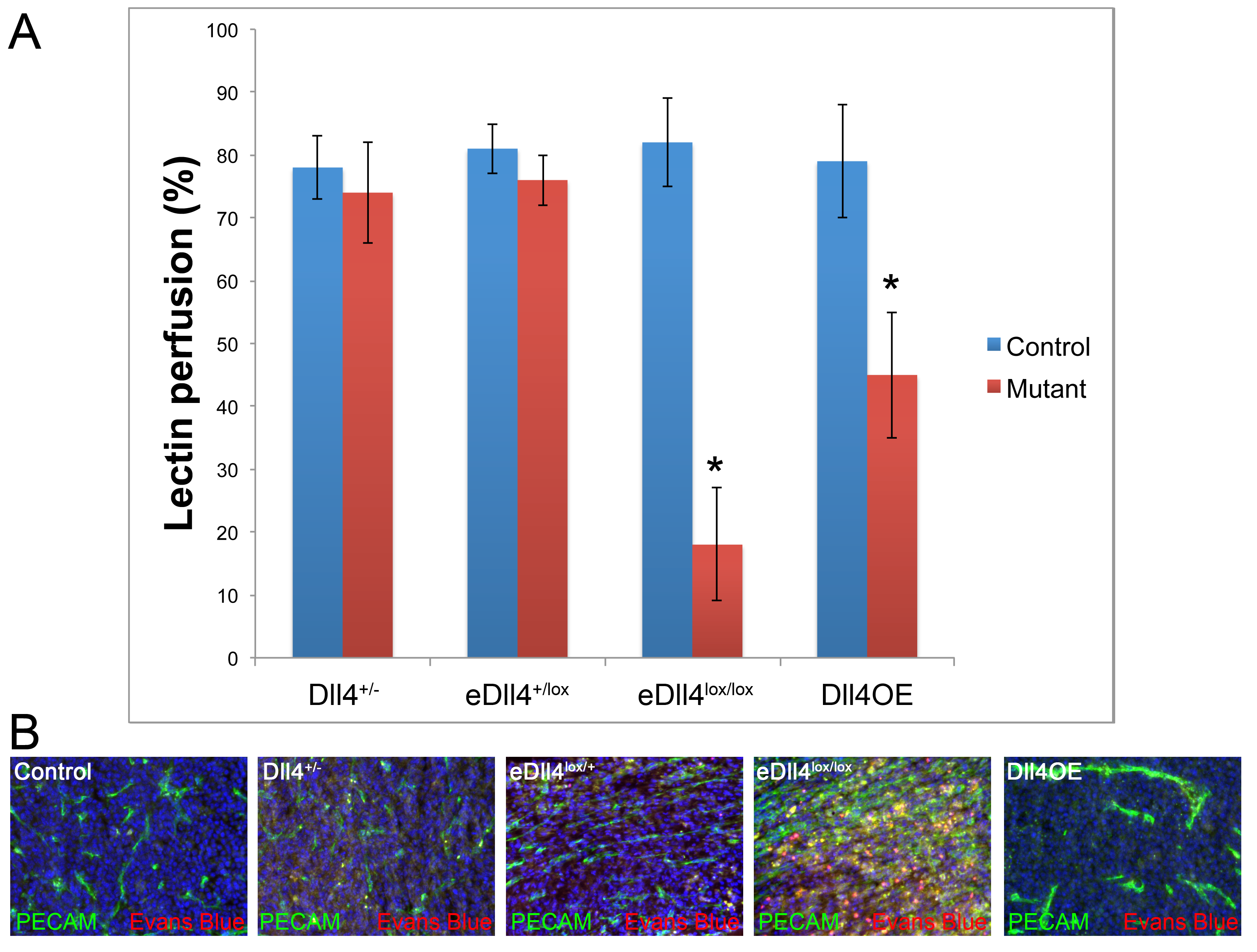

Supplement: Figure S3 — A) The percentage of lectin-perfused blood vessels is not statistically different to control levels in Dll4+/− and eDll4+/lox, while being highly reduced in eDll4lox/lox and Dll4OE. B) Evans Blue extravasation is slightly increased in Dll4 heterozygote models, highly increased in eDll4lox/lox and near normal levels in Dll4OE. * In graphics represents p<0,05. (TIF) [file pone.0029863.s003.tif]
